# A child with anorexia nervosa presenting with severe infection with cytopenia and hemophagocytosis: a case report

**DOI:** 10.1186/s13030-017-0108-6

**Published:** 2017-09-05

**Authors:** Masao Suda, Shinichiro Nagamitsu, Masahiro Kinosita, Michiko Matsuoka, Shuichi Ozono, Yasushi Otsu, Yushiro Yamashita, Toyojiro Matsuishi

**Affiliations:** 1 Department of Pediatrics and Child Health, Kurume University School of Medicine, 67 Asahi-machi Kurume City, Fukuoka, 830-0011 Japan; 2 Department of Psychiatry, Kurume University School of Medicine, Kurume, Japan

**Keywords:** Anorexia nervosa, Severe infection, Hemophagocytosis, *Acinetobacter baumannii*

## Abstract

**Background:**

Patients with anorexia nervosa in the acute phase have physical complications, such as infectious disease. Although hemophagocytic syndrome due to infection is a rare complication in anorexia nervosa, early identification for hemophagocytosis is important for avoiding a life-threatening condition.

**Case presentation:**

We report a case of a 12-year-old girl with anorexia nervosa presenting with infection with cytopenia and hemophagocytosis during initial nutritional therapy. She developed pyrexia, abdominal pain, and diarrhea during inpatient treatment. Although intravenous antibiotics were administered, the symptoms persisted. *Acinetobacter baumannii* was detected in blood culture. Hemophagocytosis was present in the bone marrow. Gamma globulin therapy was effective, with improvement in symptoms and cytopenia.

**Conclusions:**

Although our case did not fulfill the criteria of hemophagocytic syndrome, clinicians should consider severe infection in anorexia nervosa with cytopenia and hemophagocytosis.

## Background

Patients with anorexia nervosa (AN) in the acute phase have physical complications, such as bradycardia, hypotension, hypothermia, brain atrophy, and infectious disease. These patients also have life-threatening medical complications with arrhythmia, abnormal electrolyte metabolism, refeeding syndrome, and sepsis. AN can also occur in children, and early onset cases are increasing. Medical complications of AN in children are different from those in adults. Katzman reported that alterations in linear growth, impaired bone mineral accretion, and structural and functional brain changes are greater in children with AN [1]. The incidence of viral and bacterial infection is relatively uncommon in children and adult AN. However delayed recognition of infection because of a reduced or absent fever response, leads to a delay in the diagnosis of infection [2]. Brown et al. reported that changes in the immune system and cytokinesin AN occur in association with fewer symptomatic viral infections and a poorer response to bacterial infection [3]. This leads to delayed diagnosis and increased morbidity and mortality. Although hemophagocytic syndrome (HPS) due to infection is a rare complication in AN, early identification for hemophagocytosis is important for avoiding a life-threatening condition.

Infection associated with (HPS) is a life-threatening hyperinflammatory syndrome with primary symptoms of prolonged fever, cytopenia, and hemophagocytosis by activated macrophages [4]. Although the clinical course of HPS resembles sepsis, initiation of appropriate management for HPS leads to a good prognosis.

We report here a child with AN who presented cytopenia and hemophagocytosis due to *Acinetobacter baumannii* during acute management, and had successful treatment with immunoglobulin.

## Case presentation

The patient was a 12-year-old Japanese schoolgirl with weight loss. She had been healthy her entire life, and she originally had a strong sense of self-assertion and an uncompromising personality. She developed anorexia and weight loss of 20% during 2 months. She presented with a preoccupation of thinness, restriction of food intake, fearfulness of weight gain, and secondary amenorrhea. She was diagnosed as having a restricting type of AN according to the Great Ormond Street (GOS) criteria [5]. We applied the (GOS) criteria which were developed for this age. The GOS criteria are considered in younger children who rarely complain of a fear of being fat or distortion of body image. Our patient was hospitalized for treatment at our hospital and received treatment of enteral and peripheral parenteral nutrition (PPN), psychotherapy, and behavior therapy.

On admission, her height and body weight were 143.2 cm and 26.6 kg, respectively. The body mass index (BMI) and BMI standard deviation score was 13 and −3.44, respectively. The degree of obesity was −28.2%. Vital signs were almost within the normal range except for heart rate. Body temperature was 36.2 °C, blood pressure was 92/50 mmHg, respiratory rate was 18 breaths per minute, and heart rate was 60 beats per minute). Cold extremities, poor skin turgor, and hypertrichosis were observed. The patient’s blood examination at admission showed normal values except for total cholesterol (Table 1).

**Table 1:** The patient’s blood examination data at admission

	Normal value	Patient’s data
White blood cells (/μL)	3800–10,100	5000
Hemoglobin (g/dL)	11.9–14.9	13.4
Hematocrit (%)	35.0–43.0	40.3
Platelet count (/μL)	180–440×10^9^	242×10^9^
Aspartate aminotransferase (U/L)	15–30	17
Alanine aminotransferase (U/L)	9–28	11
Lactase dehydrogenase (U/L)	145–270	207
Alkaline phosphatase (U/L)	300–1380	255
Glutamyl transpeptidase (U/L)	8–34	12
Cholinesterase (U/L)	235–460	187
Total bilirubin (mg/dL)	0.3–1.1	0.68
Total protein (g/dL)	6.3–7.8	7.03
Albumin (g/dL)	3.8–4.7	4.63
Blood urea nitrogen (mg/dL)	6.8–19.2	19.4
Creatinine (mg/dL)	0.39–0.69	0.76
Uric acid (mg/dL)	2.9–6.3	5.45
Creatine kinase (U/L)	45–210	58
Sodium (mEq/L)	138–144	136
Potassium (mEq/L)	3.6–4.7	5.2
Chlorine (mEq/L)	102–109	99
Calcium (mg/dL)	8.7–10.1	9.83
Phosphorus (mg/dL)	3.6–5.8	3.92
Magnesium (mg/dL)	1.8–2.2	1.9
Copper (μg/dL)	75–102.8	95
Total cholesterol (mg/dL)	125–230	399
Triglyceride (mg/dL)	41–138	96
Free triiodothyronine (pg/mL)	2.3–4.0	0.8
Free thyroxine (ng/dL)	0.9–1.7	0.88
Thyroid-stimulating hormone (μIU/mL)	0.5–5.0	0.605

The first step of nutritional treatment consisted of a combination of oral food intake, enteral nutrition with a nasogastric tube, and PPN. After this nutritional and behavioral combination therapy, she gained weight and tube feeding was suspended. However, immediately after discontinuation of tube feeding (body weight of 28.2 kg), she developed pyrexia (39.2 °C), headache, abdominal pain, and diarrhea. There was no antecedent symptom of infection, such as a reduced fever response. A laboratory examination showed a high level of C-reactive protein (10.33 mg/dL). Although the antibiotic ceftriaxone sodium 2 g/day was administered, she still had a fever, headache, and abdominal pain.

**Fig. 1:**
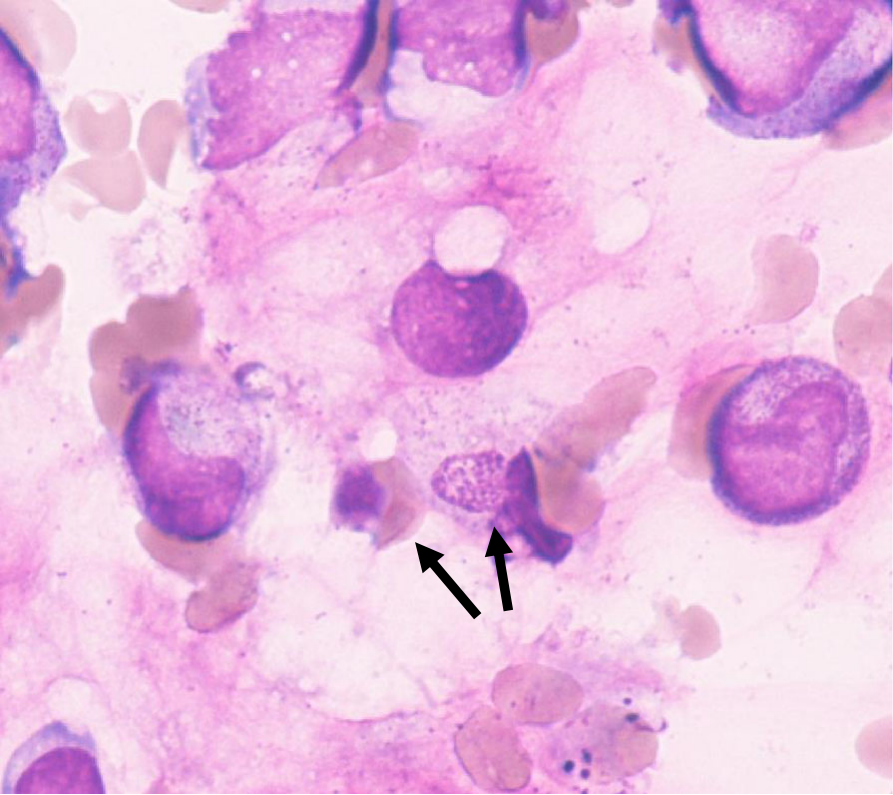
May-Giemsa staining of the bone marrow. A macrophage phagocytizing a red blood cell and granulocyte can be seen (*arrows*)

At 2 days after onset of the infectious condition, we diagnosed our patient with disseminated intravascular coagulation (DIC) because she met the diagnostic criteria for acute DIC, which included thrombocytopenia (23×10^9^ /μL), an international normalized ratio of prothrombin time of 1.26, and fibrin degradation product of 15.2 μg/mL. The neutrophil count was 651 /μL. Hemophagocytosis due to macrophages was observed in a bone marrow examination (Fig. 1). Splenomegaly was not observed in our case. The fasting triglyceride level was not higher than 265 mg/dL. The serum fibrinogen level was not lower than 150 mg/dL. Natural killer cell activity was not determined. The serum ferritin level was not higher than 500 ng/mL. The soluble interleukin-2 receptor level was not higher than 2400 U/mL. We began administration of gabexate mesilate for DIC with sepsis and changed the antibiotics to vancomycin hydrochloride and tazobactam sodium + piperacillin sodium (1:8) for the possibility of Methicillin-resistant Staphylococcus or Gram-negative bacillary infection. However, the high level of fever continued. Her hemoglobin and platelet levels declined to 10.1 g/dL and 14×10^9^ /μL, respectively, and platelet transfusion (10 units) was required. Because the albumin level declined to 2.65 g/dL, we then administered human serum albumin for 2 days. We detected *A. baumannii* in her blood culture. We started treatment with γ-globulin at 1 g/kg/dose for 1 day, which resulted in decreased fever and improvement of clinical symptoms, including the headache, abdominal pain, and diarrhea. At 4 days after administration of γ-globulin, her platelets rose to 181×10^9^ /μL.

## Discussion

Severe infection is a common complication of AN and can cause a fatal condition [6]. Our patient developed severe infection during a short disease duration. Physicians need to be aware of infection at any time of the acute and recovery phase of AN.

Immunosuppression due to malnutrition in AN is a possible cause of serious and prolonged infection. A reduced or absent fever response in AN may lead to a delay in diagnosis of infection. Hypoalbuminemia due to malnutrition may reduce the efficacy of antibiotic therapy, resulting in a prolonged febrile condition. Our patient presented with a prolonged fever response with antibiotic resistance, and she developed a severe condition that required blood transfusion.

Our case fulfilled only three (fever >38.5 °C, cytopenia, and hemophagocytosis) of eight criteria for hemophagocytic lymphohistiocytosis (HLH) [4]. Hypertriglyceridemia, hypofibrinogenemia, natural killer cell activity, and splenomegaly were not observed in our case. Our patient’s serum fibrinogen and ferritin levels were not high. Although, we could not diagnose our patient as having HLH, she might have developed HLH if we did not provide early intervention. She did not have a family history of HLH. In our case, the state of infectious hemophagocytosis was likely caused by *A. baumannii*.

Acinetobacter was identified from the patient’s blood culture as a cause of serious infection with phagocytosis. *A. baumannii* is commonly isolated from the hospital environment, and preferentially colonizes aquatic environments such as the respiratory tract in intubated patients. *A. baumannii* causes bacteremic complications in patients with an indwelling central venous catheter [7]. In our case, although the route of Acinetobacter infection was not identified, PPN or enteral feeding might have been the cause of the bacterial contamination [8, 9].

Alternatively, bacterial translocation might be a route of infection. Intestinal mucosal atrophy with long-term starvation in AN might increase the risk of bacterial translocation [10]. There are also recent reports of multidrug-resistant Acinetobacter infection in hospitals [11]. Therefore, care should be taken to reduce the risk for Acinetobacter infections as nosocomial infections in patients with AN with PPN or enteral tubes.

## Conclusions

In summary, we report a childhood case of AN where the patient presented with infection with cytopenia and hemophagocytosis during initial nutritional therapy. Regardless of HPS, clinicians should consider severe infection in AN with cytopenia and hemophagocytosis.

## Abbreviations

AN: Anorexia nervosa
BMI: Body mass index
DIC: Disseminated intravascular coagulation
HLH: Hemophagocytic lymphohistiocytosis
HPS: Hemophagocytic syndrome
PPN: Peripheral parenteral nutrition
